# Mode of Life More Important Than Sanitation

**Published:** 1914-06

**Authors:** 


**Mode of Life More Important Than Sanitation.** C. Hampton Jones, Assistant Health Officer of Baltimore, Dietetic and Hygienic Gazette, April, 1914; presents this heterodox view, basing his argument partly on the high morbidity and morality of negroes.

